# On-Ice and Off-Ice Linear Sprint Performance Across Competitive Levels in Ice Hockey Players: Insights from Continuous Velocity Profiling

**DOI:** 10.3390/sports14070290

**Published:** 2026-07-08

**Authors:** Dominik Jablonka, Aaron Uthoff, Steven Eustace, Rhys Morris, Julian Enrik Smoliga, Dusana Augustovicova

**Affiliations:** 1 Department of Physical Education & Sports Science, Setanta College, Limerick Business Complex, Keating Road, Raheen Industrial Estate, V94 Y3Y1 Limerick, Ireland; dominikjablonka37@gmail.com; 2 Sports Research Institute New Zealand (SPRINZ), School of Sport and Recreation, Auckland University of Technology, AUT-Millennium, Level 2, 17 Antares Place, Rosedale, Auckland 0632, New Zealand; aaron.uthoff@aut.ac.nz; 3 School of Human and Social Sciences, University of West London, Paragon House, Boston Manor Road, Brentford TW8 9GA, UK; steven.eustace@uwl.ac.uk; 4 School of Science and Technology, Clifton Campus, Nottingham Trent University, Nottingham NG11 8NS, UK; rhys.morris@ntu.ac.uk; 5 Department of Track & Field and Strength & Conditioning, Faculty of Physical Education and Sport, Comenius University, 81102 Bratislava, Slovakia; julian.smoliga@uniba.sk; 6 Elix Performance Center, 82105 Bratislava, Slovakia; 7 Faculty of Sports Studies, Masaryk University, Kamenice 753/5, 625 00 Brno, Czech Republic; 8 SPIRIT—Sport Performance Innovations, Research and Inclusion Team, 81102 Bratislava, Slovakia

**Keywords:** ice hockey, linear speed assessment, continuous velocity profiling, Bayesian analysis

## Abstract

Linear speed is a key performance determinant in ice hockey, yet no studies have compared continuous on-ice and off-ice velocity profiles across multiple competitive levels. This study examined continuous velocity curves and discrete sprint variables during 20 m on-ice (skating) and off-ice (running) sprints across three competitive levels. Sixty male ice hockey players (A-Team n = 20, U20A n = 20, U20B n = 20) completed maximal 20 m sprints in both conditions using a motorized resistance device. Bayesian analyses were used to evaluate continuous velocity profiles and discrete variables. Posterior estimates consistently favored greater off-ice velocity profiles than on-ice velocity profiles across all groups, particularly during the early portion of the sprint (P(diff) ~85%), although the practical meaningfulness of these differences remained uncertain. Discrete sprint variables showed stronger and more consistent practically meaningful differences (P(diff > SWC) = 66–99%). Between-group comparisons suggested a competitive hierarchy, with posterior estimates favoring greater velocity values in adults than lower competitive-level late adolescents (P(diff) ~87%). Practitioners should consider potential differences between on-ice and off-ice sprint performance and hierarchical trends across competitive levels when structuring training and assessment. Continuous velocity profiling may provide additional insight into velocity-development patterns, whereas discrete metrics may be more appropriate for routine team-level monitoring.

## 1. Introduction

Ice hockey is a high-intensity intermittent team sport in which brief sprint-skating efforts are critical for puck recovery, transitions, and offensive actions [1,2]. During short on-ice shifts, players repeatedly perform rapid accelerations interspersed with partial recoveries, and many of the highest-speed efforts in matchplay occur over distances of approximately 15–26 m [2]. Elite players have been reported to cover 484 ± 34 m per game in the sprint-skating zone above 24 km·h^−1^, while also performing frequent accelerations above 2 m·s^−2^ [1,3]. Together, these findings highlight the importance of assessing short-distance linear sprint performance in ice hockey. Previous research has shown that on-ice sprint ability differentiates players across age groups and competitive levels. Higher-level players accelerate faster than lower-level players over short skating distances, with large effect sizes reported in straight-line sprint tests [4]. Age-related differences have also been observed, with elite adult players showing faster 10 m sprint times than elite U20 players [5] and greater average velocity over the 10–20 m segment [6]. In addition, repeated linear sprint-skating ability has been moderately associated (r = 0.47) with season-long plus-minus performance in senior professionals [7]. These findings support the value of sprint testing for profiling skating performance in players from different age and competitive-level groups. 

In practice, however, on-ice testing is often limited by rink availability, scheduling, and logistical constraints, which means practitioners frequently use off-ice sprint running as a more accessible alternative for monitoring physical capacities related to skating performance [8]. This practice is partly supported by studies reporting moderate to strong relationships (r = 0.46–0.84) between off-ice and on-ice sprint outcomes in male adult, male adolescent, and female adult ice hockey players [9,10,11,12,13,14,15,16]. Nevertheless, off-ice running cannot be assumed to directly reflect skating performance. Although both are cyclical lower-limb locomotor tasks and both depend heavily on effective force production for velocity generation [17,18], skating involves greater hip abduction, external rotation, and lateral control, while the gliding phase replaces the aerial phase observed in running [19,20,21]. From acceleration to maximal velocity, key temporal characteristics also differ in an opposite manner, with sprint skating showing increasing contact times and decreasing step rate, whereas sprint running demonstrates decreasing contact times and increasing step rate [22]. These mechanical differences suggest that similar outcomes between off-ice and on-ice tests may not necessarily reflect similar velocity-generation strategies.

Despite these insights and widespread testing practices, an important limitation remains in how sprint performance has been evaluated. Several studies have focused on discrete sprint outcomes such as split times, average velocity over fixed distances, or total sprint time. While useful in practice, such measures provide limited insight into how velocity develops across the sprint [23]. This is important because strong associations or similar split times may still coexist with meaningful differences in velocity-development patterns between running and skating, particularly during acceleration [24]. Recently, Glaude-Roy et al. [25] reported large associations (r = −0.71) between maximal theoretical horizontal velocity derived from on-ice sprint skating and off-ice sprint-running performance. However, the use of discrete variables to quantify sprint velocity, such as split times over fixed distances (e.g., 0–5 m, 5–10 m), may overlook important information or key differences, particularly when comparing on-ice and overground sprinting. 

Continuous velocity profiling through the use of motorized resistance devices (MRDs) may help address the limitation of solely using discrete variables. A MRD is a tethered system capable of continuously measuring position, velocity, force, and power during locomotor tasks, while simultaneously applying controlled resistance or assistance [26]. As such, it enables sprint velocity to be quantified across the entire sprint distance. Continuous velocity profiling should not necessarily be viewed as a replacement for traditional discrete sprint metrics, but rather as a complementary analytical approach that may provide additional insight into how velocity develops throughout sprint performance. This approach allows for the identification of differences between overground and on-ice sprinting conditions throughout the sprint, rather than at isolated points. In contrast, discrete measures such as total sprint time or split times may fail to detect phase-specific differences, as they are dependent on a priori variable selection and do not reflect how velocity develops across the sprint [27]. By analyzing the full velocity–distance profile, it becomes possible to identify differences at any point in the sprint and to better characterize distinct phases such as acceleration, peak velocity, and deceleration [28]. This may be particularly important given the biomechanical differences between overground running and ice skating during the acceleration phase. Consequently, continuous velocity profiling may provide additional insight into how velocity is generated under different conditions. 

Furthermore, combining continuous profiling with traditional discrete metrics may offer a more comprehensive assessment of sprint performance, with practical relevance for performance monitoring and exercise prescription, as well as for understanding differences between players across developmental and competitive levels [29,30]. Therefore, the aim of the present study was to compare on-ice and off-ice 20 m sprint performance across competitive levels using continuous velocity profiles, including comparisons between conditions within each level and between levels within each condition. A secondary aim was to perform the same comparisons using traditional discrete sprint variables to examine whether discrete and continuous approaches provide similar or complementary insights into sprint performance. Based on the biomechanical differences between running and skating, it was hypothesized that off-ice sprinting would demonstrate greater sprint velocities than on-ice sprinting across the sprint distance. Given previous evidence showing superior sprint performance in older and higher-level ice hockey players, it was further hypothesized that sprint performance would follow a competitive-level hierarchy in both testing conditions.

## 2. Materials and Methods

### 2.1. Study Design

This study used an observational cross-sectional design with repeated measurements under two testing conditions. The main study factors were condition (on-ice vs. off-ice; within-subject) and group (A-Team, U20A, U20B; between-subject). The primary outcomes were continuous sprint velocity profiles across the 20 m sprint and discrete sprint performance variables, including split times, average segment velocities, peak velocity, and distance to peak velocity. Data were collected from a single ice hockey club comprising three teams that differed in both age and competitive level. The order of team testing and participants within each team was randomized on each testing day. However, testing conditions could not be randomized due to ice-rink scheduling constraints.

### 2.2. Participants

A total of 60 male ice hockey players (20.31 ± 6.18 years; 183.17 ± 5.86 cm; 82.81 ± 9.75 kg) from three teams participated in this study: the elite adults team (A-Team) (n = 20; 26.64 ± 6.94 years; 183.90 ± 6.20 cm; 88.52 ± 8.59 kg; 20.86 ± 6.51 years of ice hockey experience), the elite U20 team (U20A) (n = 20; 17.26 ± 1.19 years; 183.68 ± 5.57 cm; 81.26 ± 8.81 kg; 11.26 ± 1.85 years of ice hockey experience), and the sub-elite U20 team (U20B) (n = 20; 16.61 ± 1.20 years; 181.78 ± 5.85 cm; 77.78 ± 8.93 kg; 10.56 ± 1.42 years of ice hockey experience). Eligible participants were male ice hockey players from the A-Team, U20A, and U20B squads who were medically cleared for full participation and regularly engaged in team training and competition at the time of testing. Players were excluded if they were goalkeepers, had any current injury or medical complaint that limited full participation in training or maximal sprinting, had not been regularly participating in team training in the week prior to testing, or had incomplete data due to technical or procedural issues. Additional exclusion criteria included engaging in strenuous physical activity within 24 h before testing or consuming a full meal within two hours prior to testing, while light snacking was permitted. The study was approved by the Ethics Committee of Institute of Sport Science at University of Würzburg (approval no. EV2023/5-1608) and conducted in accordance with the Declaration of Helsinki. Written informed consent was obtained from all participants or, in the case of participants under 18 years of age, from their legal guardians.

### 2.3. Procedures

Testing was conducted across two consecutive days, with skating performance assessed on Day 1 and running performance assessed on Day 2, ensuring a minimum recovery period of 24 h between sessions. All testing was performed using the MRD (1080 Sprint, Sprint 1; 1080 Motion AB, Västerås, Sweden), operated via its dedicated software interface. The device functioned in non-flying-weight mode, applying a minimal isotonic load of 1 kg [31]. For each participant and condition, the best trial was retained for analysis. For subsequent analysis, both raw and general data exports were obtained from the 1080 Motion software (Web App 1.0; 1080 Motion AB, Västerås, Sweden). The raw data export included continuous velocity and position metrics sampled at a frequency of 333 Hz, whereas the general data export contained pre-calculated parameters generated by the system’s internal algorithms. 

#### 2.3.1. Linear 20 m Off-Ice Sprint Running Test

**Figure 1:**
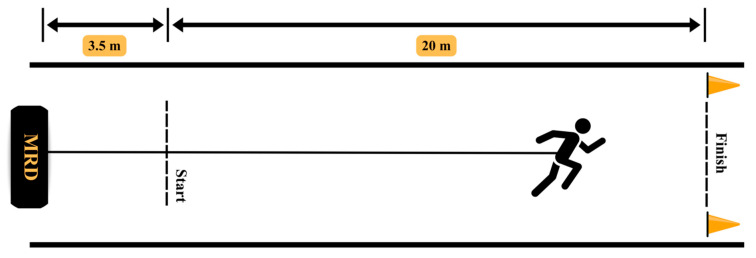
Linear 20 m off-ice sprint running test.

The test (Figure 1) was conducted in an indoor stadium with a one-centimetre-thick rubber surface laid over concrete. MRD was positioned 3.5 m behind the starting line, with the tether attached centrally to the dorsal pelvic belt at the level of the sacrum using a carabiner. Before testing, participants completed a standardized warm-up. Two cones were placed 20 m from the starting line to indicate the finish line. Athletes began from a static two-point staggered stance and initiated the sprint at their own discretion following the instruction: “You can start whenever you feel ready.” The trial was automatically triggered once the system detected a movement velocity exceeding 0.2 m·s^−1^, minimizing the possibility of false starts caused by minor tether motion. Each player performed two maximal-effort 20 m sprints, with a recovery period of three to four minutes between trials to minimize fatigue and preserve sprint performance [32]. Trials involving slipping, stumbling, obvious pacing, premature deceleration before the finish line, or technical malfunction of the MRD system were excluded and repeated after the same standardized recovery period. Raw velocity data were recorded in real-time using the 1080 Motion software for subsequent analysis.

#### 2.3.2. Linear 20 m On-Ice Sprint Skating Test

**Figure 2:**
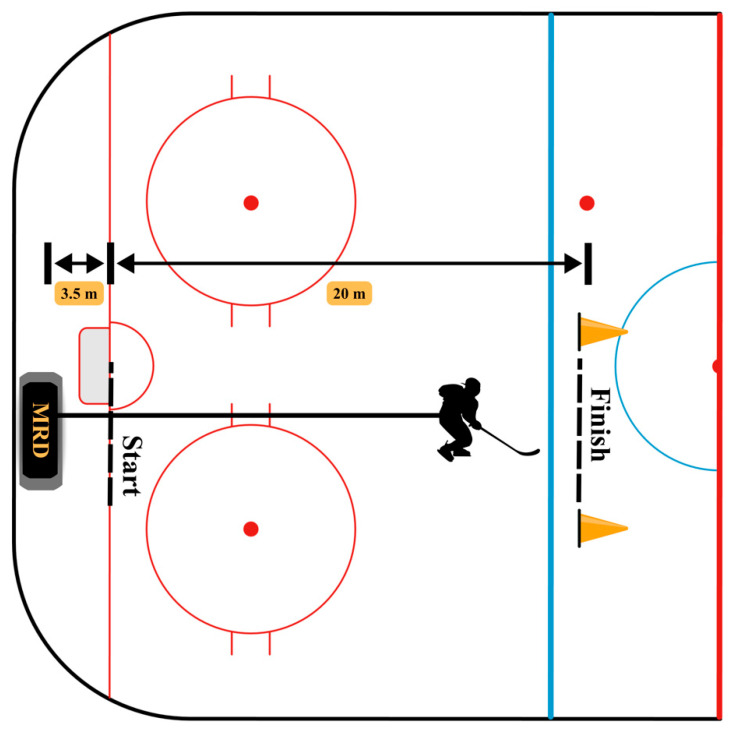
Linear 20 m on-ice sprint skating test.

On the following day, the linear 20 m on-ice sprint skating test (Figure 2) was performed. The setup replicated the off-ice test to ensure comparability between conditions. MRD was positioned 3.5 m behind the starting line, placed on a protective rubber mat in front of the end boards and glass. As in the off-ice test, the tether was attached to the participant’s pelvic belt at the level of the sacrum using a carabiner. Before testing, players completed a standardized warm-up. Two cones were positioned at the 20 m mark to indicate the finish line. Participants started from a static forward-facing stance on the goal line, replicating the split-stance foot position used in the off-ice trial. The front skate was externally rotated to simulate the initial off-ice push-off mechanics without crossover and maintain consistency across test conditions. Athletes initiated each sprint skating trial at their own discretion following the instruction: “You can start whenever you feel ready.” MRD automatically triggered data recording once a movement velocity greater than 0.2 m·s^−1^ was detected, ensuring standardized trial initiation. Each athlete completed two maximal-effort trials, with a recovery period of three to four minutes between attempts to minimize fatigue and preserve sprint performance [32]. Trials involving slipping, stumbling, unintended crossover steps, premature deceleration before crossing the finish line, loss of tether tension, or technical malfunction of the MRD system were excluded and repeated following the same standardized recovery interval. All data were recorded in real time using the 1080 Motion software for subsequent velocity analysis.

### 2.4. Data Processing and Outcome Derivation

Raw sprint velocity data were exported from the MRD and processed in R Statistical Software (version 4.6.1; R Foundation for Statistical Computing, Vienna, Austria). As participants completed the 20 m sprint in varying times and, therefore, generated a different number of raw data points, velocity data from each trial were normalized to 0–100% of sprint distance to enable comparison of velocity-development patterns independent of sprint duration. This allowed direct comparison of continuous velocity values at corresponding relative portions of the sprint across testing conditions and groups.

In addition to the continuous velocity profiles, discrete sprint outcomes were derived from the device export. These included split times and average velocities over 0–5 m, 5–10 m, 10–15 m, and 15–20 m, as well as peak velocity and the distance at which peak velocity was achieved. These derived outcomes were used to complement the continuous velocity analysis and to allow comparison with commonly used sprint performance metrics.

### 2.5. Statistical Analysis

For the discrete sprint outcomes, Bayesian hierarchical regression models were fitted to estimate differences according to condition (on-ice vs. off-ice), group (A-Team, U20A, U20B), and the relevant within-group and between-group comparisons. Participant ID was included as a random effect to account for repeated measurements within individuals. For the continuous velocity profiles, Bayesian spline-based models (thin-plate splines) were used to analyze velocity across the normalized sprint distance. This approach enabled comparison of velocity curves between conditions and across groups while accounting for repeated observations within participants. Weakly informative priors were used to regularize parameter estimation, including a Student-t prior to improve robustness to outliers. Posterior distributions were summarized using posterior means and 95% highest density intervals (HDIs). To aid interpretation, the posterior probability that a difference exceeded zero (P(diff)) and the posterior probability that a difference exceeded the smallest worthwhile change (SWC) (P(diff > SWC)) were calculated. The SWC was defined as 0.2 times the within-group standard deviation [33]. All analyses were performed in R using the brms package [34], and model convergence was evaluated using standard diagnostic procedures, including R-hat values and posterior predictive checks [35].

## 3. Results

### 3.1. Between Conditions Within Group (Off vs. On-Ice Velocity Curves)

**Figure 3:**
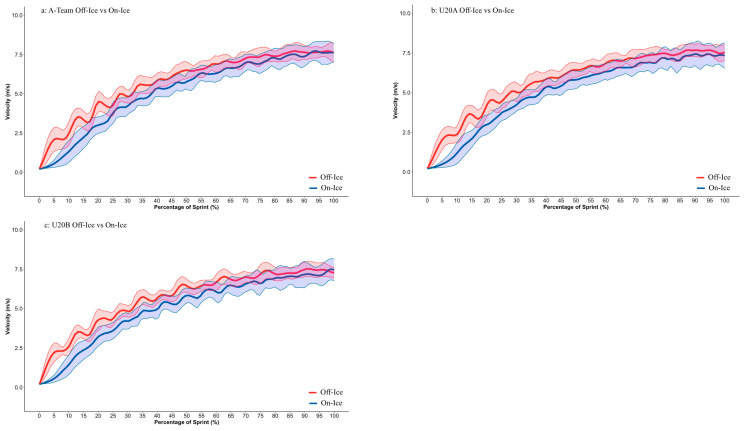
Continuous velocity profiles comparing on-ice and off-ice sprint conditions across groups. (**a**) A-Team, (**b**) U20A, and (**c**) U20B. Raw velocity curves are presented for both conditions, normalized to the percentage of the sprint distance. Solid lines represent the mean velocity profiles, and the shaded regions represent the standard deviation around the mean.

**Figure 4:**
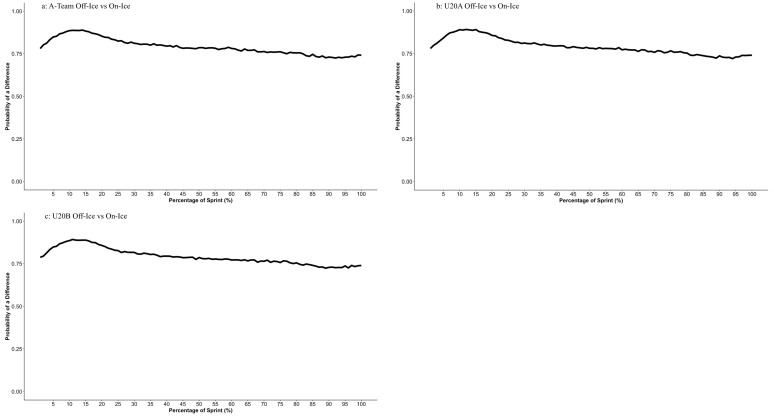
Posterior probability of a difference displaying if first condition (off-ice) achieved greater velocities than the second condition (on-ice). (**a**) A-Team, (**b**) U20A, and (**c**) U20B. Posterior probabilities are presented across the percentage of the sprint.

**Figure 5:**
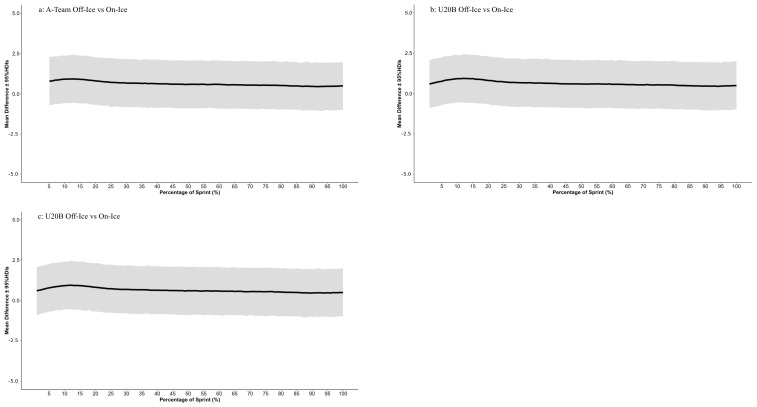
Mean velocity difference between the first condition (off-ice) and the second condition (on-ice), with 95% highest density intervals (HDI) displayed across the percentage of the sprint. (**a**) A-Team, (**b**) U20A, and (**c**) U20B.

Off- and on-ice between condition comparisons of the sprint-velocity curve are presented in Figure 3. The early portion of the sprint (0–15% of total distance) demonstrated the highest posterior probability of a difference between conditions (~85% P(diff)), with posterior estimates favoring greater off-ice velocity compared with on-ice velocity across all groups. Across the remainder of the sprint-velocity curve, posterior estimates continued to favor greater off-ice velocity across the remainder of the sprint, with P(diff) values of approximately 75% (Figure 4). However, uncertainty surrounded these estimates, as the mean differences with 95% HDIs consistently crossed zero across the continuous velocity curve (Figure 5). Furthermore, the probability that differences exceeded the smallest worthwhile change remained low (~11% P(diff > SWC)) across all comparisons.

### 3.2. Within Condition Between Groups (A-Team vs. U20A vs. U20B Velocity Curves)

**Figure 6:**
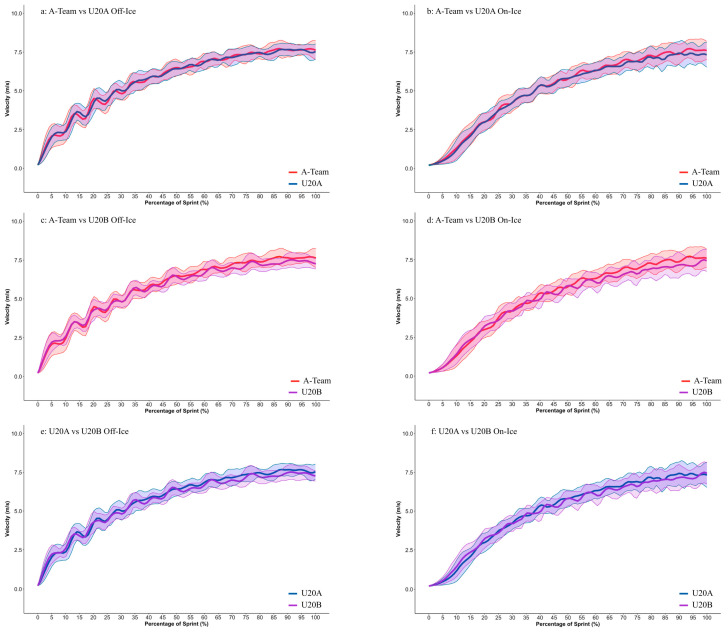
Continuous velocity profiles comparing groups in on-ice and off-ice conditions. (**a**) A-Team vs. U20A off-ice, (**b**) A-Team vs. U20A on-ice, (**c**) A-Team vs. U20B off-ice, (**d**) A-Team vs. U20B on-ice, (**e**) U20A vs. U20B off-ice, and (**f**) U20A vs. U20B on-ice. Raw velocity curves are presented for both conditions, normalized to the percentage of the sprint distance. Solid lines represent the mean velocity profiles, and the shaded regions represent the standard deviation around the mean.

**Figure 7:**
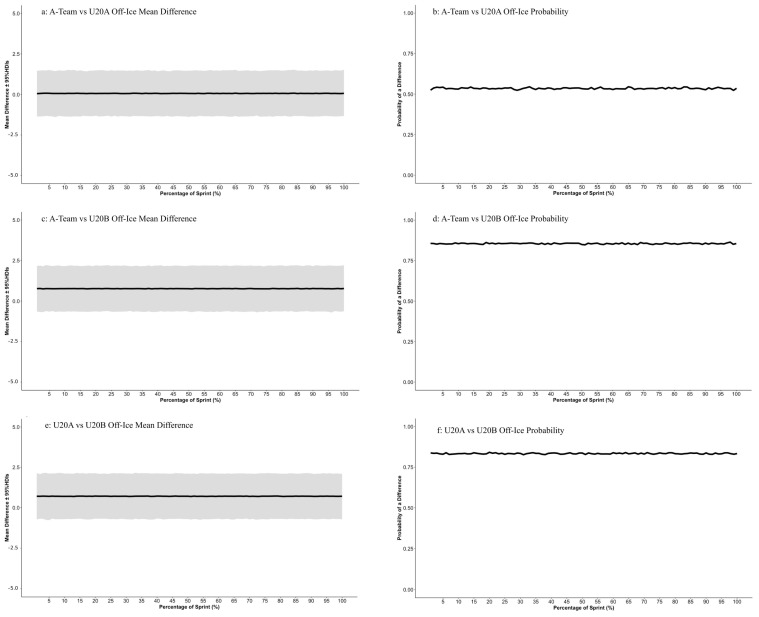
Off-ice mean velocity differences between groups with 95% highest density intervals (HDI) and posterior probabilities indicating whether the first group achieved greater velocities than the second group. (**a**) A-Team vs. U20A mean difference, (**b**) A-Team vs. U20A probability, (**c**) A-Team vs. U20B mean difference, (**d**) A-Team vs. U20B probability, (**e**) U20A vs. U20B mean difference, and (**f**) U20A vs. U20B probability.

**Figure 8:**
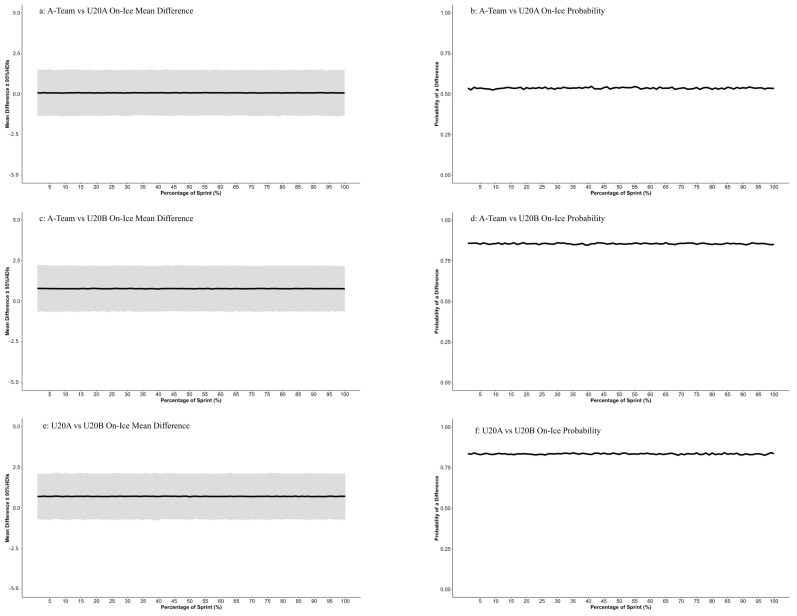
On-ice mean velocity differences between groups with 95% highest density intervals (HDI) and posterior probabilities indicating whether the first group achieved greater velocities than the second group. (**a**) A-Team vs. U20A mean difference, (**b**) A-Team vs. U20A probability, (**c**) A-Team vs. U20B mean difference, (**d**) A-Team vs. U20B probability, (**e**) U20A vs. U20B mean difference, and (**f**) U20A vs. U20B probability.

Between-group comparisons of the continuous velocity curve for both conditions are shown in Figure 6 (a,c,e for off-ice; b,d,f for on-ice). Across the off-ice velocity curves (Figure 7b,d,f), posterior estimates tended to favor greater velocities in the A-Team relative to U20A (P(diff) ≈ 61%) and U20B (P(diff) ≈ 88%). Likewise, posterior estimates favored greater velocities in U20A compared with U20B (P(diff) ≈ 83%). Similar directional tendencies were observed under on-ice conditions (Figure 8b,d,f), with P(diff) of ~63%, ~86%, and ~81% for A-Team vs. U20A, A-Team vs. U20B, and U20A vs. U20B, respectively. However, these directional tendencies should be interpreted cautiously, as the mean differences with 95% HDIs consistently crossed zero across all sprint-velocity curves (Figure 7a,c,e and Figure 8a,c,e), suggesting uncertainty in the results. Moreover, P(diff > SWC) was approximately ~11% across the sprint–velocity curve for on-ice group comparisons. For off-ice sprint-velocity curves between groups, the probability of observing a P(diff > SWC) was also approximately ~11% for comparisons between A-Team and U20A, and between U20A and U20B. However, the P(diff > SWC) was ~47% for comparisons between A-Team and U20B in off-ice condition.

### 3.3. Discrete Variables Comparisons

The analysis of discrete variables between off- and on-ice sprint conditions (within-group; Table 1) revealed that posterior estimates consistently favored greater off-ice velocities compared with on-ice velocities across all groups. Posterior probabilities favored off-ice performance, with a 66–99% P(diff) that off-ice exceeded on-ice, and a 72–99% P(diff > SWC). Conversely, time-related variables were consistently slower on-ice, indicating longer sprint durations across all groups (73–99% P(diff) favoring longer on-ice times; 66–98% P(diff > SWC)). Mean differences whose 95% HDIs did not include zero were observed for time-based metrics in the 0–5 m segment (A-Team, U20A), 5–10 m segment (all groups), 10–15 m segment (U20A, U20B), and total 0–20 m time (U20A). The distance at which peak velocity was achieved tended to be greater on-ice (56–66% P(diff)), suggesting that maximal skating velocity was reached later than in off-ice running, albeit with uncertainty. Between-group comparisons within each condition (Table 2) demonstrated a consistent directional trend across velocity-related metrics (A-Team > U20A > U20B). This directional pattern was observed in both on-ice and off-ice conditions, although differences were greater on-ice (on-ice velocity: 50–79% P(diff) A-Team > U20B; off-ice velocity: 50–80% P(diff)). Time-based metrics showed similar directional tendencies; however, the probability of practically meaningful differences remained relatively low (P(diff > SWC): 16–47%).

## 4. Discussion

Although off-ice sprint testing is frequently used as a practical alternative to on-ice assessment in ice hockey, limited research has examined whether running and skating demonstrate similar velocity-development patterns throughout an entire sprint. Previous studies have relied primarily on discrete sprint outcomes, which may overlook differences in how velocity develops throughout the sprint. Accordingly, the primary aim of this study was to compare 20 m sprint velocity characteristics between off- and on-ice conditions and to examine differences between groups differing in age and competitive level (A-Team, U20A, U20B). A secondary aim was to compare continuous velocity profiling with traditional discrete sprint metrics to determine whether these approaches provide similar or complementary insights into sprint performance. This is the first study to analyze 20 m continuous velocity curves across off- and on-ice conditions in multiple competitive-level groups using an MRD. The present approach enabled examination of velocity-development patterns throughout the sprint across both conditions and competitive levels. The findings indicated that the early acceleration phase (0–15% of sprint distance) showed the highest posterior probability of a difference between off- and on-ice velocity curves (~85% P(diff)), with posterior estimates favoring off-ice performance. However, the practical meaningfulness of these differences remained uncertain, given that 95% HDIs crossed zero and P(diff > SWC) remained low across the sprint. Between-group comparisons showed directional trends aligned with competitive hierarchy (A-Team > U20A > U20B) across both on-ice and off-ice conditions, although similar uncertainty was observed for these comparisons. For discrete variables, split times provided the clearest evidence of between-condition differences, whereas velocity-based metrics generally showed greater uncertainty. Similarly, between-group comparisons were characterized by uncertainty despite directional trends favoring the expected competitive hierarchy. Taken together, these findings suggest that continuous velocity profiling and traditional discrete metrics provide complementary information regarding sprint performance. While split times appeared more sensitive for detecting between-condition differences, continuous velocity profiling provided additional insight into how velocity developed throughout the sprint and where differences between conditions or competitive levels emerged. 

### 4.1. Between Conditions Within Group (Off vs. On-Ice Velocity Curves)

Off- vs. on-ice comparisons indicated a tendency toward greater off-ice velocity across all groups, with the highest posterior probability of a difference occurring during the early sprint phase (0–15% of total distance) (~85% P(diff)). The tendency toward greater velocities during the early phase of the off-ice sprints may reflect environmental constraints rather than athlete-specific limitations. Specifically, the reduced friction of the ice compared with off-ice surfaces limits horizontal force application, whereas off-ice surfaces allow greater force production through the shoe-ground interaction, supporting greater initial acceleration. Across the remainder of the sprint, posterior estimates continued to favor greater off-ice velocity, with P(diff) values of approximately 75%. This directional trend was consistent with our a priori hypothesis that off-ice sprinting would favor greater velocity development than on-ice skating. Off-ice running allows athletes to apply greater ground reaction forces with each step, supported by greater friction coefficients [17]. In contrast, skating requires a lateral push-off, and due to the low-friction blade-ice interface [30,36], a smaller proportion of the total force is directed in the forward sprint direction during initial acceleration, resulting in a more gradual increase in velocity [37]. The forward start technique used in the on-ice test may also contribute to these differences. Many players typically initiate on-ice acceleration with a crossover start in game situations, which can facilitate more immediate velocity generation by increasing blade contact and enabling more effective forward-directed force application during the initial steps. Although speculative, this difference in starting strategy could affect early acceleration. Nevertheless, the gradual acceleration in continuous velocity curves observed here aligns with previous research using similar forward-start protocols [36,38], suggesting that technique plays a role but does not entirely explain the discrepancy. The observed greater off-ice velocities, particularly during the early acceleration phase, must be interpreted with caution given the uncertainty in our analyses and the limited evidence of a practically meaningful difference (P(diff > SWC) ≈ 11%). Although the observed between-condition differences were directionally consistent with established biomechanical principles, the present study extends previous literature by applying continuous velocity profiling and Bayesian curve-based analyses to characterize velocity development throughout the sprint. From a practical standpoint, while off-ice running generally produces greater velocities than on-ice skating, the meaningful difference across conditions remains uncertain. This comparison should, therefore, be interpreted cautiously, as the mechanical demands differ between conditions, including previously noted variations in ground reaction forces and friction coefficients [19,22]. Off-ice testing remains a practical and accessible method for evaluating neuromuscular qualities, particularly when ice time is limited [8]. However, on-ice testing remains essential for capturing sport-specific mechanical and technical characteristics that cannot be replicated through running-based assessments [24]. Performance interpretation should therefore account for individual technique, training background, and testing context rather than assuming a fixed relationship between on-ice and off-ice performance. Both conditions may offer complementary insights, with the present study’s continuous velocity profiling approach providing a novel method to identify phase-specific characteristics that discrete measures inherently aggregate.

### 4.2. Within-Condition Between Groups (A-Team vs. U20A vs. U20B Velocity Curves)

Between-group comparisons of sprint-velocity curves suggested a hierarchical pattern (A-Team > U20A > U20B) across both off- and on-ice conditions. Under off-ice conditions, the probability that A-Team outperformed U20A was ~61%, increasing to ~88% versus U20B, while U20A showed ~83% probability of greater velocity than U20B. Similar trends were observed on-ice (~63%, ~86%, ~81% P(diff) for the same comparisons). This expected hierarchical pattern aligns with our a priori hypothesis and with theoretical differences in training experience and neuromuscular development [39]. Consistent with the implications discussed in the previous section, these results further illustrate that continuous velocity profiling can refine the characterization of between-group differences across the acceleration phase, capturing nuances that may not be evident from discrete split times alone. When used in conjunction with traditional split times, velocity curves offer practitioners a more comprehensive view of sprint performance, enabling identification of specific phases where competitive-level differences are most pronounced. Elite adults (A-Team) have accumulated more sport-specific training and technical refinement compared to younger age groups, with differences in training status, training history, and competitive match demands providing a basis for performance differences between groups [5]. Prior research has shown that older and higher-level ice hockey players achieve faster sprint split times and greater average velocities [5,6], and the present study extends these findings by suggesting that these advantages may be present across the full velocity curve rather than only at discrete splits. These findings should be interpreted with caution given the uncertainty in our estimates, as evidenced by the small probabilities of practically meaningful differences (P(diff > SWC) ≈ 11% across most curves), with the only notable exception being the off-ice comparison between the A-Team and U20B group (P(diff > SWC) ≈ 47%). For more robust athlete profiling, velocity curve analysis should be combined with discrete performance metrics, technical assessments, and longitudinal monitoring to capture changes not apparent in a single 20 m sprint. The similar hierarchical patterns across both on-ice and off-ice conditions indicate that these relationships are consistent regardless of condition, though magnitude may vary slightly depending on the biomechanical demands of each environment.

### 4.3. Discrete Variables Comparisons

The analysis of discrete sprint variables between conditions indicated that time-based metrics provided relatively high posterior probability of a difference. For the 0–5 m segment, A-Team and U20A had 98% P(diff). Similarly, the 5–10 m split showed strong evidence for a difference with 96–98% P(diff). Later segments (10–15 m) also showed strong evidence of differences for U20A and U20B (98% P(diff)) but not for A-Team. Total 0–20 m time showed the most certain difference only for U20A (98% P(diff)). Although previous studies have shown moderate to large (r = 0.54–0.81) correlations between on-ice and off-ice discrete sprint variables [9,10], the present results suggest that these relationships should be interpreted cautiously, as discrete time-based differences between conditions were evident despite greater uncertainty in velocity-based measures. In contrast, velocity-based variables (average speeds, top speed, distance to peak velocity) showed more modest differences (66–99% P(diff)), although HDIs consistently crossed zero, indicating greater uncertainty compared with time-based metrics. The only exception with more robust certainty was average speed 5–10 m in U20B (99% P(diff); HDIs not crossing zero). Therefore, our results suggest that split times may provide greater sensitivity for detecting between-condition differences than continuous velocity profiling. Nevertheless, this observation is consistent with the secondary aim of the study, which was to compare continuous velocity profiling and traditional discrete sprint metrics in order to examine whether these approaches provide similar or complementary insights into sprint performance. 

Whereas discrete metrics appeared more sensitive for identifying overall between-condition differences, continuous velocity profiling provided greater detail regarding the continuous velocity development throughout the sprint. These patterns mirror the continuous velocity curves, but split times showed markedly greater certainty compared with the continuous curve analysis. One possible explanation for the greater certainty observed in discrete metrics is that split times aggregate performance across a larger segment of the sprint, thereby reducing the influence of moment-to-moment variability in velocity development. In contrast, continuous velocity profiling preserves individual fluctuations in velocity throughout the sprint, which may increase uncertainty around point-by-point estimates despite similar overall performance outcomes. Consequently, small phase-specific differences may accumulate into clearer differences in discrete split times while remaining less certain when evaluated continuously across the entire velocity curve. This may explain why discrete metrics demonstrated stronger evidence of between-condition differences than continuous velocity profiles, particularly during the later phases of acceleration. While split time differences align with previous research [15] and were confirmed here through Bayesian analysis, the uncertainty observed in continuous velocity profiling suggests considerable individual variation that discrete measures may obscure, offering practitioners additional information regarding individual velocity-development patterns. Therefore, the practical value of continuous velocity profiling may lie less in detecting overall between-condition differences and more in characterizing how velocity develops throughout the sprint. Between-group comparisons within each condition followed the expected hierarchical trend (A-Team > U20A > U20B), evident in both on-ice and off-ice conditions, albeit with uncertainty (HDIs constantly crossing zero). Velocity metrics provided stronger evidence for hierarchical differences than time metrics, particularly between the most disparate groups (A-Team vs. U20B). P(diff) approached 80% for most velocity discrete variables (except Average Speed 0–5 m), whereas time-based metrics rarely exceeded 50%.

### 4.4. Methodological Considerations and Limitations

This study is not without limitations. The present sample size, while comparable to similar ice hockey studies [14,16], resulted in greater uncertainty in between-group estimates, particularly when comparing adjacent competitive levels. Furthermore, continuous velocity-profile analyses involve point-by-point estimation across the sprint and may, therefore, require larger sample sizes than traditional discrete analyses to identify small but potentially meaningful differences with greater certainty. Future studies with larger samples may help reduce uncertainty, improve the precision of continuous velocity-profile estimates, and determine whether the directional trends observed in the present study represent practically meaningful differences across the sprint. In addition, positional distribution (e.g., forwards and defensemen) was not considered in the present analyses. Although this was beyond the scope of the present study and would have substantially reduced statistical power due to small subgroup sample sizes, positional demands may influence sprint-performance characteristics [2] and should be explored in future research. Furthermore, age, playing experience, and competitive level were inherently intertwined within the present sample, and, therefore, their independent contributions to sprint performance could not be determined. Both on-ice and off-ice assessments provide valuable but distinct information, with off-ice testing offering reduced environmental variability [26] and on-ice testing capturing sport-specific technical demands. In addition, testing order was fixed, with on-ice testing performed on Day 1 and off-ice testing on Day 2, which may have introduced order, familiarization, or residual fatigue effects. Although participant order was randomized within each testing day, testing conditions could not be counterbalanced due to logistical constraints associated with ice-rink availability. Nevertheless, on-ice testing remains valuable for capturing sport-specific demands, but the findings should be interpreted in the context of this methodological limitation. Future studies should seek to implement randomized or counterbalanced testing orders where feasible in order to further reduce the potential influence of order, familiarization, and learning effects. Additionally, future research should compare forward and crossover start techniques to determine whether crossover starts enable more rapid early velocity generation through a pre-tilted skate position and more horizontally directed force application, compared with forward starts where effective push-off is delayed by the need for external hip rotation. The present findings suggest that sprint performance alone may be insufficient to differentiate athletes across age groups and competitive levels. However, sprint testing may be useful as part of a broader assessment battery that includes strength, power, technical skill, and game-based metrics [40]. Additionally, discrete metrics provide support for routine monitoring, whereas continuous velocity profiling offers deeper insight into specific sprint phases, enabling targeted interventions [28]. Finally, when performance differences are uncertain (small P(diff), small P(diff > SWC), or HDIs crossing zero), practitioners should acknowledge this uncertainty and avoid over-interpreting small differences from a single assessment. Longitudinal monitoring across multiple sessions, contextualized within training cycles and combined with complementary performance indicators, may provide a more robust foundation for informed decisions.

## 5. Conclusions

Continuous velocity profiling indicated a tendency toward greater off-ice sprint velocities than on-ice sprint velocities, particularly during early acceleration, together with a hierarchical pattern across competitive levels (A-Team > U20A > U20B). However, the practical meaningfulness of these differences remained uncertain. Discrete sprint variables, particularly split times, provided the clearest evidence of between-condition differences, whereas between-group comparisons within each condition remained largely uncertain. From a practical perspective, continuous velocity profiling may offer additional insight into velocity-development patterns and individual variability during linear sprinting that may not be apparent from discrete metrics alone. For routine team-level monitoring, discrete metrics may therefore be more appropriate, while continuous velocity profiling may be used to inform individualized assessment of velocity-development characteristics. Off-ice sprint testing may be particularly useful in less skilled or developing cohorts, whereas its ability to differentiate athletes at higher competitive levels may be reduced, highlighting the continued importance of on-ice assessments. Accordingly, off-ice and on-ice sprint testing should be viewed as complementary components of comprehensive performance profiling that integrates sprint performance with strength, power, technical, and game-based metrics. Continuous velocity profiling may provide complementary information regarding sprint-velocity development when used alongside traditional discrete sprint metrics.

## Figures and Tables

**Table 1:** Between-condition (within-group) comparisons for discrete variables.

Group	Metric	Mean On-Ice	SD On-Ice	Mean Off-Ice	SD Off-Ice	Probability of a Difference (%)	95% HDI (Lower to Upper)	Probability of a Difference > SWC (%)
A-Team	Average Speed 0–5 m (m/s)	3.22	0.32	3.94	0.31	95	−1.57 to 0.11	97
U20A		3.16	0.33	3.92	0.33	96	−1.62 to 0.09	97
U20B		3.23	0.32	3.92	0.33	94	−1.52 to 0.16	96
A-Team	Average Speed 5–10 m (m/s)	6.14	0.24	6.43	0.24	86	−0.83 to 0.25	90
U20A		6.07	0.26	6.39	0.26	89	−0.86 to 0.21	92
U20B		5.84	0.24	6.43	0.24	99	−1.12 to −0.06	99
A-Team	Average Speed 10–15 m (m/s)	7.07	0.26	7.30	0.26	83	−0.69 to 0.25	88
U20A		6.90	0.30	7.27	0.30	94	−0.84 to 0.09	97
U20B		6.73	0.35	7.10	0.34	94	−0.83 to 0.09	97
A-Team	Average Speed 15–20 m (m/s)	7.36	0.29	7.58	0.29	75	−0.84 to 0.41	81
U20A		7.21	0.32	7.42	0.32	75	−0.83 to 0.41	81
U20B		7.50	0.25	7.67	0.25	70	−0.79 to 0.47	75
A-Team	Top Speed 0–20 m (m/s)	7.58	0.31	7.73	0.31	66	−0.87 to 0.57	72
U20A		7.37	0.35	7.69	0.34	82	−1.06 to 0.39	84
U20B		7.26	0.35	7.45	0.36	70	−0.91 to 0.51	76
A-Team	Distance to Top Speed (m)	17.73	1.43	16.87	1.45	66	−3.02 to 4.82	61
U20A		17.24	1.56	16.91	1.56	56	−3.54 to 4.28	50
U20B		17.79	1.52	17.12	1.52	63	−3.21 to 4.65	57
A-Team	Split Time 0–5 m (s)	1.59	0.12	1.28	0.12	98	0.01 to 0.62	97
U20A		1.62	0.13	1.28	0.13	98	0.03 to 0.65	98
U20B		1.58	0.12	1.30	0.12	97	−0.02 to 0.59	96
A-Team	Split Time 5–10 m (s)	0.83	0.03	0.78	0.03	97	0.00 to 0.09	96
U20A		0.86	0.03	0.80	0.03	99	0.01 to 0.10	98
U20B		0.83	0.03	0.78	0.03	97	0.00 to 0.09	96
A-Team	Split Time 10–15 m (s)	0.71	0.03	0.69	0.03	86	−0.02 to 0.06	80
U20A		0.73	0.03	0.69	0.03	98	0.00 to 0.08	96
U20B		0.75	0.03	0.71	0.03	98	0.00 to 0.08	96
A-Team	Split Time 15–20 m (s)	0.67	0.02	0.65	0.02	73	−0.03 to 0.06	66
U20A		0.68	0.03	0.66	0.03	83	−0.02 to 0.07	76
U20B		0.70	0.03	0.67	0.03	84	−0.02 to 0.07	76
A-Team	Total Time 0–20 m (s)	3.83	0.21	3.41	0.21	96	−0.06 to 0.91	94
U20A		3.91	0.23	3.42	0.22	98	0.01 to 0.99	97
U20B		3.90	0.21	3.48	0.20	95	−0.07 to 0.90	94

Note: A-Team = adult elite group; U20A = Under-20 elite group; U20B = Under-20 sub-elite group; m/s = meters per second; m = meters; s = seconds; SD = standard deviation; HDI = highest density interval; SWC = smallest worthwhile change. Probability values represent Bayesian posterior probabilities of between-condition differences. For clarity, probability values for velocity metrics were inverted so that greater values reflect faster off-ice performance compared to on-ice performance. Probabilities of a difference > SWC and 95% HDIs are reported in the original orientation.

**Table 2:** Between-group (within-condition) comparisons for discrete variables.

Comparison	Metric	Probability of a Difference (%)	95% HDI (Lower to Upper)	Probability of a Difference > SWC (%)
On-Ice A-Team vs. U20A	Average Speed 0–5 m (m/s)	56	−0.84 to 0.93	50
On-Ice A-Team vs. U20B		50	−0.89 to 0.88	45
On-Ice U20A vs. U20B		45	−0.97 to 0.83	40
Off-Ice A-Team vs. U20A		53	−0.86 to 0.93	47
Off-Ice A-Team vs. U20B		53	−0.84 to 0.94	47
Off-Ice U20A vs. U20B		50	−0.92 to 0.91	45
On-Ice A-Team vs. U20A	Average Speed 5–10 m (m/s)	58	−0.55 to 0.70	52
On-Ice A-Team vs. U20B		79	−0.44 to 1.03	76
On-Ice U20A vs. U20B		73	−0.51 to 0.96	68
Off-Ice A-Team vs. U20A		54	−0.64 to 0.73	48
Off-Ice A-Team vs. U20B		50	−0.67 to 0.68	44
Off-Ice U20A vs. U20B		46	−0.78 to 0.70	41
On-Ice A-Team vs. U20A	Average Speed 10–15 m (m/s)	67	−0.58 to 0.87	62
On-Ice A-Team vs. U20B		77	−0.74 to 1.14	74
On-Ice U20A vs. U20B		64	−0.72 to 1.12	59
Off-Ice A-Team vs. U20A		52	−0.73 to 0.79	46
Off-Ice A-Team vs. U20B		68	−0.73 to 0.78	59
Off-Ice U20A vs. U20B		63	−0.70 to 0.96	63
On-Ice A-Team vs. U20A	Average Speed 15–20 m (m/s)	65	−0.60 to 0.87	60
On-Ice A-Team vs. U20B		75	−0.63 to 1.09	70
On-Ice U20A vs. U20B		64	−0.74 to 1.02	58
Off-Ice A-Team vs. U20A		59	−0.66 to 0.82	53
Off-Ice A-Team vs. U20B		74	−0.57 to 0.83	30
Off-Ice U20A vs. U20B		64	−0.75 to 1.04	69
On-Ice A-Team vs. U20A	Top Speed 0–20 m (m/s)	69	−0.67 to 1.11	63
On-Ice A-Team vs. U20B		74	−0.73 to 1.30	70
On-Ice U20A vs. U20B		59	−0.94 to 1.12	54
Off-Ice A-Team vs. U20A		53	−0.89 to 0.94	48
Off-Ice A-Team vs. U20B		80	−0.88 to 0.93	77
Off-Ice U20A vs. U20B		67	−0.65 to 1.19	68
On-Ice A-Team vs. U20A	Distance to Top Speed (m)	59	−3.62 to 4.67	53
On-Ice A-Team vs. U20B		48	−4.15 to 3.98	43
On-Ice U20A vs. U20B		41	−4.79 to 3.92	35
Off-Ice A-Team vs. U20A		50	−4.13 to 4.15	44
Off-Ice A-Team vs. U20B		45	−4.34 to 3.92	39
Off-Ice U20A vs. U20B		45	−4.54 to 4.05	39
On-Ice A-Team vs. U20A	Split Time 0–5 m (s)	66	−0.36 to 0.32	38
On-Ice A-Team vs. U20B		47	−0.31 to 0.34	47
On-Ice U20A vs. U20B		42	−0.31 to 0.37	52
Off-Ice A-Team vs. U20A		51	−0.35 to 0.34	44
Off-Ice A-Team vs. U20B		55	−0.34 to 0.31	40
Off-Ice U20A vs. U20B		54	−0.36 to 0.32	41
On-Ice A-Team vs. U20A	Split Time 5–10 m (s)	56	−0.09 to 0.06	38
On-Ice A-Team vs. U20B		79	−0.11 to 0.07	19
On-Ice U20A vs. U20B		70	−0.11 to 0.06	26
Off-Ice A-Team vs. U20A		55	−0.08 to 0.07	39
Off-Ice A-Team vs. U20B		72	−0.09 to 0.06	24
Off-Ice U20A vs. U20B		63	−0.10 to 0.07	33
On-Ice A-Team vs. U20A	Split Time 10–15 m (s)	70	−0.09 to 0.05	25
On-Ice A-Team vs. U20B		79	−0.11 to 0.08	19
On-Ice U20A vs. U20B		66	−0.10 to 0.07	29
Off-Ice A-Team vs. U20A		60	−0.07 to 0.06	35
Off-Ice A-Team vs. U20B		73	−0.09 to 0.07	37
Off-Ice U20A vs. U20B		66	−0.09 to 0.06	22
On-Ice A-Team vs. U20A	Split Time 15–20 m (s)	67	−0.08 to 0.05	28
On-Ice A-Team vs. U20B		78	−0.10 to 0.06	20
On-Ice U20A vs. U20B		65	−0.09 to 0.07	30
Off-Ice A-Team vs. U20A		81	−0.08 to 0.06	16
Off-Ice A-Team vs. U20B		81	−0.09 to 0.54	16
Off-Ice U20A vs. U20B		65	−0.09 to 0.07	31
On-Ice A-Team vs. U20A	Total Time 0–20 m (s)	62	−0.67 to 0.49	32
On-Ice A-Team vs. U20B		60	−0.65 to 0.51	35
On-Ice U20A vs. U20B		49	−0.54 to 0.57	45
Off-Ice A-Team vs. U20A		51	−0.63 to 0.59	43
Off-Ice A-Team vs. U20B		61	−0.64 to 0.49	33
Off-Ice U20A vs. U20B		60	−0.63 to 0.50	34

Note: A-Team = adult elite group; U20A = Under-20 elite group; U20B = Under-20 sub-elite group; m/s = meters per second; m = meters; s = seconds; HDI = highest density interval; SWC = smallest worthwhile change. Probability values represent Bayesian posterior probabilities of between-group differences. For clarity, probability values for time metrics were inverted so that greater values indicate faster performance in the more competitive/older group. Probabilities of a difference > SWC and 95% HDIs are reported in the original orientation.

## Data Availability

The data that support the findings of this study are available from the corresponding author upon reasonable request.
